# Ultralow-loss geometric phase and polarization shaping by ultrafast laser writing in silica glass

**DOI:** 10.1038/s41377-020-0250-y

**Published:** 2020-02-04

**Authors:** Masaaki Sakakura, Yuhao Lei, Lei Wang, Yan-Hao Yu, Peter G. Kazansky

**Affiliations:** Optoelectronics Research Centre, University of Southampton, Southampton, SO17 1BJ UK

**Keywords:** Laser material processing, Metamaterials

## Abstract

Polarization and geometric phase shaping via a space-variant anisotropy has attracted considerable interest for fabrication of flat optical elements and generation of vector beams with applications in various areas of science and technology. Among the methods for anisotropy patterning, imprinting of self-assembled nanograting structures in silica glass by femtosecond laser writing is promising for the fabrication of space-variant birefringent optics with high thermal and chemical durability and high optical damage threshold. However, a drawback is the optical loss due to the light scattering by nanograting structures, which has limited the application. Here, we report a new type of ultrafast laser-induced modification in silica glass, which consists of randomly distributed nanopores elongated in the direction perpendicular to the polarization, providing controllable birefringent structures with transmittance as high as 99% in the visible and near-infrared ranges and >90% in the UV range down to 330 nm. The observed anisotropic nanoporous silica structures are fundamentally different from the femtosecond laser-induced nanogratings and conventional nanoporous silica. A mechanism of nanocavitation via interstitial oxygen generation mediated by multiphoton and avanlanche defect ionization is proposed. We demonstrate ultralow-loss geometrical phase optical elements, including geometrical phase prism and lens, and a vector beam convertor in silica glass.

## Shaping light: Nanopores offer control with lower losses

The optical property of birefringence can be manipulated in silica glass using an ultrafast laser writing procedure, creating materials in which the phase and polarization of light can be controlled with significantly lower scattering losses than alternative techniques. Birefringence is the effect observed when the refractive index of a material varies depending on the polarization and direction of incident light. M. Sakakura, Y. Lei, L. Wang, Y.-H. Yu, and P. Kazansky at the University of Southampton, UK, used laser light with pulses in the femtosecond range to create elongated nanopores inside silica glass. When the nanopores are aligned perpendicular to incident polarized light they allow subtle control of the light’s properties with transmission as high as 99%. The ability of the materials to “shape” visible, near-infrared and ultra-violet light is expected to be beneficial in a variety of specialist optical applications.

## Introduction

Conventional optics (e.g. lenses or mirrors) manipulate the phase via optical path difference by controlling the thickness or refractive index of the material. Recently, a promising type of optics has emerged that exploits the geometric phase (GP) shift when a lightwave is transformed by a parameter other than optical path difference, e.g. polarization. The wavefront is modified by introducing spatially varying anisotropy and is a result of the GP, also known also as Pancharatnam–Berry phase^1,2^. For example, when circularly polarized light is transmitted through a half-wave plate, the light experiences an additional phase shift, which is proportional to twice the rotation angle of the waveplate. This property enables encoding of desired phase profiles by writing birefringence with different optical axis orientations in birefringent materials. Theoretically any phase pattern can be achieved solely by means of the GP with efficiencies reaching 100%^3^.

Fabrication of polarization GP optical elements (GPOEs) requires birefringence patterning^3–7^. Plasmonic meta-surfaces produced by lithographic methods enable thin optical elements with high spatial resolution^5,6^. However, the challenge is achieving meta-surfaces with high transmission and high efficiency in a visible range. Although various GPOEs can be fabricated by photoalignment of liquid crystals^3,4^, their applications are limited by the high absorption in the UV and infrared regions, and low thermal and chemical durability.

Birefringence patterning technologies have also been used for producing light beams with space-variant polarization known as vector beams^8^. In particular, vector beams presenting cylindrical symmetry have been demonstrated with liquid crystals by means of electrically tuned q-plates^9^. Radial vector beams are especially interesting due to the non-vanishing longitudinal electric field component presented in tightly focusing systems, which allows one to sharply focus light below the diffraction limit^10^. This property has been of great significance in fields, such as laser machining^11^, plasmonic focusing^8^, molecular orientation determination^12^ and particle acceleration^13^. On the other hand, azimuthal vector beams can induce longitudinal magnetic fields with potential applications in spectroscopy and microscopy^14^. In addition, vector beams have other interesting applications in fundamental science^15–17^.

Femtosecond laser writing^18–22^ has emerged as a technologically attractive method for birefringence patterning via the formation of polarization-controlled self-assembled lamella structures (nanogratings, referred as type II modification) in various bulk and thin film transparent materials and silica glass in particular^23–31^. GPOEs and vector beam polarization converters (S-waveplates) have been fabricated by polarization control of the nanograting orientation and the related optical axis of the form birefringence^23^. In particular, silica glass is an ideal optical material due to its wide transmission window, high chemical and thermal durability and high optical damage threshold^32^. However, a drawback of birefringent modification produced by nanogratings is the reduced transmission, especially in the visible and ultraviolet, which originates from fluctuations of the periodicity in the nanograting structures^24,28^. A higher transmission, of ~90% at 532 nm, can be achieved by delivering a higher density of incident pulses per μm of scan path at a low writing speed, which allows the formation of more uniform nanogratings^24,33^. However, the losses are still significantly high compared with conventional optical elements due to the scattering loss, especially in the UV region. The cross section of Rayleigh scattering is proportional to the sixth power of the size of nanostructures, indicating that reducing the size of the anisotropic structures could further reduce the loss.

Here, we report a new type of femtosecond laser-induced birefringent modification originating from randomly distributed nanopores in silica glass, which provides ultralow scattering loss with 99% transmission in the visible range and higher than 90% transmission in the UV spectral range down to 330 nm. Imaging of the structures with scanning electron microscopy (SEM) reveals the formation of nanopores elongated perpendicular to the polarization, which are responsible for the birefringence. We demonstrate a GP prism, a GP lens and a polarization convertor with ultrahigh transmission in a broad spectral range. The technology of low-loss polarization and GP patterning widens the applications of GPOEs and vector beam convertors for high power lasers and visible and UV light sources^34^.

## Results

### Optical properties and structures of ultralow-loss birefringent modification

The formation of uniform nanogratings is key to reducing the optical loss in birefringent modifications in silica glass^24,33^. Typically, more uniform nanogratings require a larger number of laser pulses (or a higher pulse density, *N*_d_) or writing at a slower scanning speed, which is the normal strategy to reduce the optical loss. Here, in contrast, we discovered that lower optical loss could be obtained by producing random nanostructures in silica with a smaller number of pulses.

**Fig. 1:**
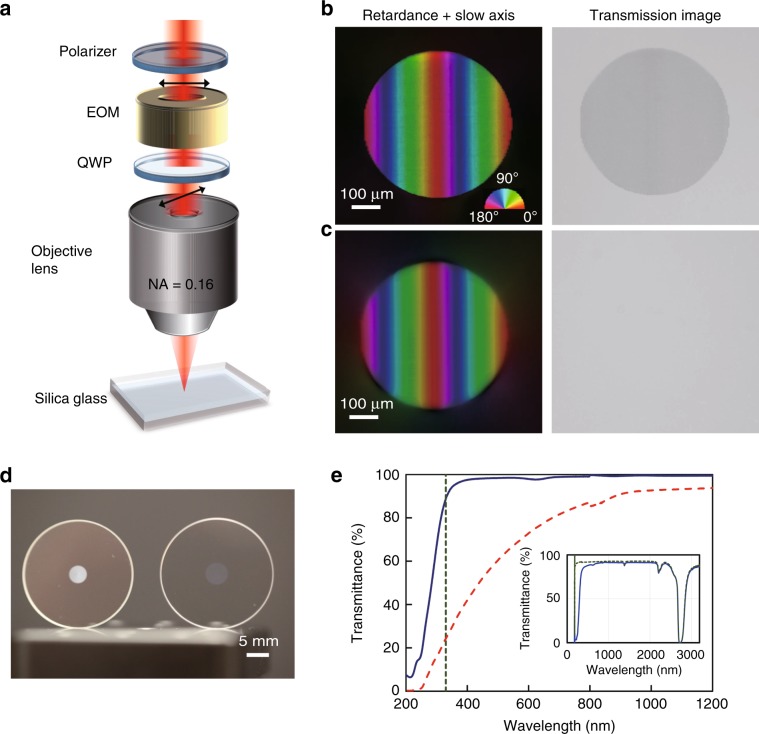
Laser writing of birefringence structures inside silica glass. **a** Illustration of patterning of birefringence in silica glass by femtosecond laser direct writing. **b** and **c** Birefringence and transmission optical microscope images of birefringence structures with high-loss (type II) and low-loss modifications (type X), respectively. To obtain comparable retardance, one birefringent layer was written for **b**, while 10 birefringent layers were written for **c**. The colour scale in **b** indicates the slow axis of the birefringence. **d** Photograph of birefringent optics imprinted with high-loss (left) and low-loss (right) modifications in silica glass plates. **e** Transmission spectra of birefringent structures of high-loss (red dashed line) and low-loss (blue solid line) modifications with reference to pristine silica glass. The vertical dotted line indicates a wavelength of 330 nm, at which the transmission for the low-loss modification is 90%. The inset shows the transmission spectra of the low-loss modification (blue solid line) and pristine silica glass (green dashed line)

A birefringent pattern was created by writing parallel lines with an interline separation of 1 μm (raster scanning) (Fig. 1a) using the writing parameters (300 fs, 0.7 μJ, 3.7 J/cm^2^, 12 TW/cm^2^, 200 kHz, 1 mm/s corresponding to 200 pulses/μm) that generate the conventional birefringent modification originating from nanogratings (Fig. 1b). The imprinted pattern with a birefringence of 2.1 × 10^−3^ can be clearly observed in the transmitted light without any polarization optics (Section 2 of Supplementary). Additionally, a birefringent pattern with negligible transmission loss was obtained by writing at a faster scanning speed of 6 mm/s, corresponding to a lower pulse density of 33 pulses/μm, with the other parameters unchanged (Fig. 1c). The imprinted birefringent pattern was almost invisible in the transmitted light, regardless of the retardance (~150 nm with 10 layers) being comparable with that of the modification produced by nanogratings (Fig. 1b). The birefringence of the modification was 6 × 10^−4^ while maintaining high transparency (Section 2 of Supplementary).

The transmission difference between the high-loss and low-loss birefringent modifications was also apparent with the naked eye and from the measurement of their transmission spectra (Fig. 1d, e, respectively). For the high-loss modification, the transmittance in the near-infrared region was ~90%, but it sharply decreased in the visible region. On the other hand, for the low-loss modification, the transmittance was as high as 99% in the visible and near-infrared spectral regions (400−2400 nm) and higher than 90% in the UV spectral range down to 330 nm. This difference indicates that the low-loss birefringent modification, referred to as type X, is caused by a formation mechanism fundamentally different from the conventional one^34^. The low-loss modification has a weak absorption band at ~620 nm, which has been attributed to the non-bridging oxygen hole centre (NBOHC). This absorption can be easily quenched by annealing at 400 °C for 2 h^35^ (Fig. S5c of Supplementary)

**Fig. 2:**
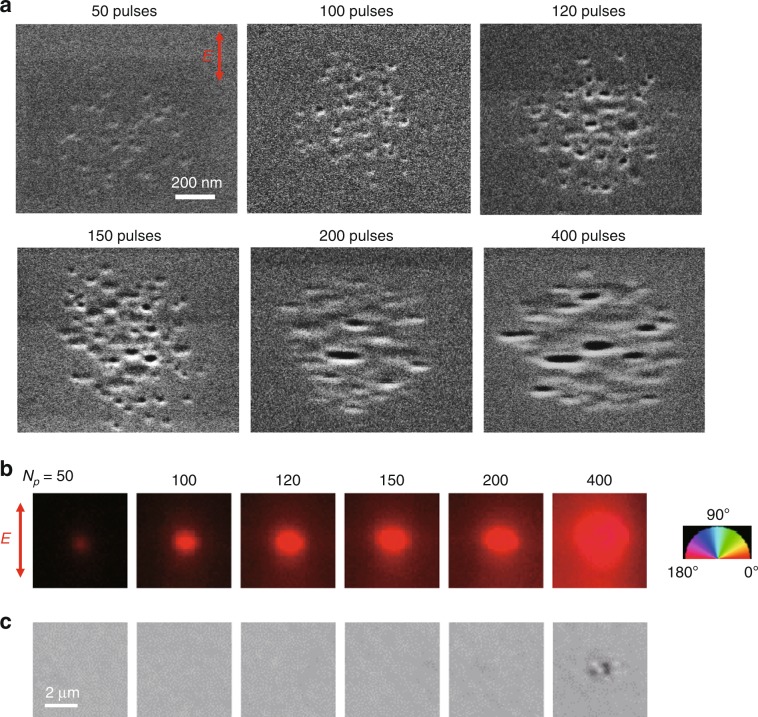
Microstructures of birefringent modifications written with different numbers of pulses. **a** SEM images of the polished surfaces of modifications written by laser irradiation (300 fs, 0.7 μJ) with different pulse numbers (*N*_p_). **b** and **c** Retardance and optical transmission images of laser-induced modifications with different pulse numbers, respectively. The red colour in **b** means that the slow axis of the induced birefringence is parallel to the horizontal direction. In the transmission images, the modification was visible at *N*_p_ ≥ 200, and elongation of the modification was observed at *N*_p_ *=* 400

To explore the origin of the low-loss birefringent modification and its evolution with the number of pulses (*N*_p_), SEM images of the structures were obtained (Fig. 2a) and compared with their birefringence and optical transmission images (Fig 2b, c). The SEM image of the structure at *N*_p_ = 50 showed the appearance of randomly distributed nanopores (Fig. 2a), which produced birefringence with a small retardance of ~1 nm and the slow axis oriented perpendicular to the polarization of the writing beam (Fig. 2b). With increasing pulse number, the density of nanopores increased and the shape was elongated perpendicular to the polarization direction (*N*_p_ = 120–150). At the same time, the retardance of the induced birefringence also increased with the increase of the nanopore density (birefringence of ~5 × 10^−4^ at *N*_p_ *=* 150). Importantly, the slow axis of the birefringence was always parallel to the elongation of the nanopores. The morphology of the nanopores experienced a change around *N*_p_ = 200, corresponding to the formation of nanoplanes followed by their self-assembly, at which the modification was visible (Fig. 2c, *N*_p_ ≥ 200). The transition from the low-loss to high-loss modification with the formation of nanoplanes indicates that the smaller size of the nanostructures could be responsible for the high transmittance.

Another interesting observation in the SEM and retardance images (Fig. 2a, b) is that the diameter of the modification of 1 μm is much smaller than that of the beam spot at the focus, which is ~5 μm. The smaller diameter indicates that the modification occurs only in the central part of the focused beam due to multiphoton absorption, in which at least eight 1030 nm photons are necessary for the band gap of silica glass (9 eV)^36^. The size of the modification being smaller than the beam spot diameter is a feature of type X not typical for the modification with nanogratings which are commonly produced with high NA lenses generating modifications comparable with the beam spot diameter in the focus.

### Factors for the formation of low-loss birefringent modifications

**Fig. 3:**
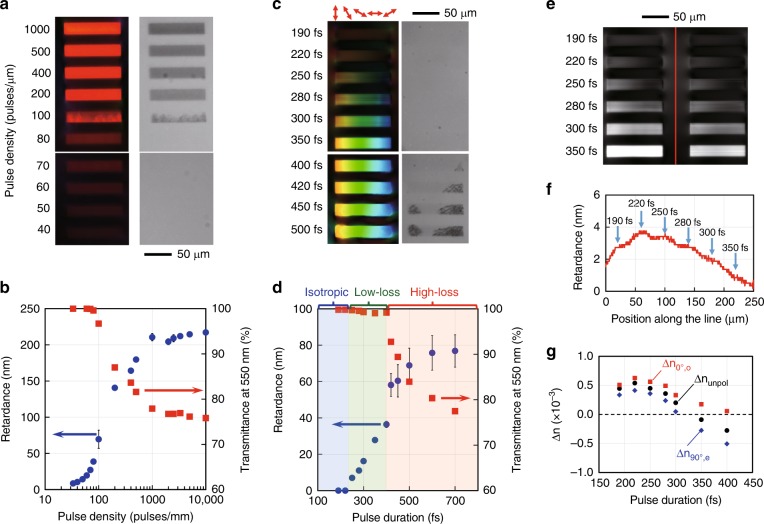
Birefringence and transmission of laser written patterns with different parameters. **a** and **c** Retardance (left) and transmission (right) images of birefringent structures written at different pulse densities and pulse durations, respectively. In **c**, the polarization was rotated during laser writing. **b** and **d** Plots of the retardance (blue circles) and transmittance (red squares) of the modified regions in **a** and **c** against the pulse density and pulse duration, respectively. In **d**, the ranges of the pulse duration for different modification types are indicated by different colours. **e** Retardance distribution in the area of birefringent structures written with different pulse durations. **f** Retardance plotted along the dashed line in **e**, where stress is formed between the laser written structures. **g** Refractive index change in the modified regions written with different pulse durations (Section 3 of Supplementary). The refractive index was measured using a phase imaging camera. Δ*n*_unpol_, Δ*n*_0°,o_ and Δ*n*_90°,e_ are the refractive index changes measured with unpolarized light and with horizontally (0°) and vertically (90°) polarized light. The slow axis of birefringence in the modified region is horizontal (0°). The small difference of Δ*n*_0°,o_ > Δ*n*_90°,e_ at a shorter pulse duration is due to stress-induced birefringence

There are several crucial factors for the formation of low-loss birefringent modifications. The first factor is the pulse density or number of pulses (Figs. 1 and 2). To more clearly observe the transition from the low-loss (type X) to high-loss (type II) modifications, the birefringent structures written by raster scanning were investigated as functions of the pulse density and pulse duration (Fig. 3). With increasing pulse density, the retardance increased and the modification abruptly became visible in the transmitted light at *N*_d_ = 100 pulses/μm (Fig. 3a). The dependences of the retardance and transmittance on the pulse density (Fig. 3b) showed a drop in the transmission and a sharp increase in the retardance at the same pulse density (*N*_d_ = 100 pulses/μm). These sharp changes indicate that there is no intermediate state between the low-loss and high-loss modifications.

The second crucial factor is the laser pulse duration (*t*_p_) (Fig. 3c, d). Modifications of different types^30,37^ appeared with changing pulse duration. For pulse durations shorter than 250 fs, the modification was highly transparent, but birefringence that depends on the polarization of the writing light was not observed. The isotropic modifications at *t*_p_ < 250 fs had a positive refractive index change, which is due to compaction of the silica structure (referred to as type I)^38^. With increasing pulse duration, birefringence that depends on the polarization appeared while maintaining high transmittance. Clear transmission loss became visible in the transmission image at *t*_p_ ≥ 400 fs, corresponding to the generation of the high-loss modification (type II). A more detailed investigation showed that no low-loss birefringent modification was observed when the pulse duration was shorter than 220 fs or longer than 500 fs (Section 1 of Supplementary). This limitation of the pulse duration for the low-loss birefringent modification suggests a different mechanism from that of nanograting formation.

Interestingly, the stress around the laser written regions depended on the pulse duration (Fig. 3e, f). The compressive stress-induced birefringence in the vicinity of the laser written region was the largest at the pulse duration of 220 fs, at which isotropic modification due to glass compaction occurred. In contrast, the stress-induced birefringence became almost negligible at 350 fs, at which the birefringence of the low-loss modification is almost maximum. This suggests that the expansion due to the formation of nanopores and the compaction of the glass structure was balanced, resulting in an almost negligible net volume change at 350 fs. The balance of the compaction and void formation in the low-loss birefringent modification was verified by refractive index change measurements (Fig. 3g, Section 3 of Supplementary for more detail). The refractive index change was positive at a shorter pulse duration (~5 × 10^−4^ at 220 fs), which indicates densification of the silica structure in the laser written region. With increasing pulse duration, the refractive index change decreased while the difference between the two refractive indices for two orthogonal polarizations, i.e., the birefringence (Δ*n*_0°,o_ − Δ*n*_90°,e_), increased. This opposite behaviour of refractive index change and birefringence indicates that the number of nanopores increased in the densified glass structures with increasing pulse duration, making negligible net volume change possible at certain laser writing parameters. Similar negligible volume expansion due to the balance between glass densification and nanopore formation has also been reported in different laser irradiation conditions^39^.

Other crucial factors are the numerical aperture (NA) and wavelength (Section 4 of Supplementary). We found that the formation of low-loss birefringent modifications (type X) becomes more difficult with higher NA. Moreover, no type X modification was observed with NA > 0.30 and by photoexcitation for a 515 nm wavelength writing beam. No type X modification at higher NAs and shorter wavelengths suggests that the size of the photoexcited volume might influence the formation dynamics of nanopores. For example, focusing with a higher NA makes the spot size smaller, which generates a higher energy density at the threshold fluence for modification in silica glass. The higher energy density easily overcomes the tensile strength of the material (Young’s modulus), which prevents the formation of spatially separated nanopores. The detailed mechanism is still the subject of current investigation, which will be reported elsewhere.

## Discussion

### Origins of low transmission loss and birefringence

**Fig. 4:**
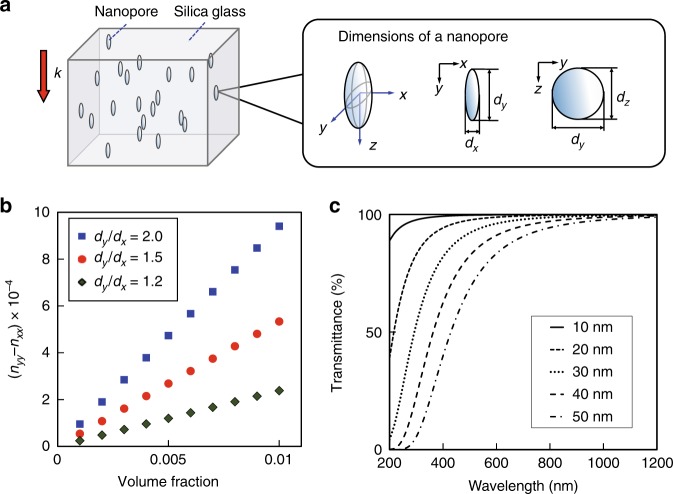
Simulation of optical properties of the low-loss birefringent modification. **a** Model for calculation of the birefringence. Oblate nanopores are distributed in silica glass. **k** indicates the light propagation direction for evaluation of the birefringence. **b** Plot of the birefringence (*n*_*yy*_ − *n*_*xx*_) against the volume fraction of nanopores with different aspect ratios (*d*_*y*_*/d*_*x*_). **c** Optical transmission spectra of silica glass with nanopores of different diameters simulated based on Rayleigh scattering. The volume fraction of nanopores for the simulation is *f* = 0.005, and the thickness of the volume in which nanopores are formed is 400 μm, which is the same as that of the birefringent structure in Fig. 1c

The comparison between the SEM images and optical images clearly shows the correlation between the emergence of nanoplanes and the transmission drop, which suggests that the randomly distributed nanopores is responsible for the reduced light scattering. Moreover, the oblate shape of nanopores should be responsible for the induced birefringence. To confirm this, we simulated the transmittance and birefringence of the randomly distributed nanopore structures in silica glass (Fig. 4a).

The birefringence (*n*_*yy*_ − *n*_*xx*_) originating from oblate nanopores inside silica glass was simulated as a function of the aspect ratio of the nanopores (*d*_*y*_/*d*_*x*_) and the volume fraction of nanopores (*f*) in silica glass (Fig. 4a, b) by the Maxwell–Garnett model with anisotropic scattering particles (Section 5 of Supplementary)^40^. The birefringence is proportional to the volume fraction of nanpores and the slope increases with the aspect ratio of the nanopores. The birefringence (*n*_*yy*_ − *n*_*xx*_) is always positive for *d*_*y*_/*d*_*x*_ > 1, meaning that the slow axis is parallel to the long axis of the nanopores, which is consistent with the relation between the slow axis and the elongation of the nanopores in the SEM images (Fig. 2a, b). To explain the observed birefringence of 5 × 10^−4^ at *N*_p_ = 150 (Fig. 2a), the volume fraction of nanopores should be ~0.5% (*f* = 0.005) for mean aspect ratio *d*_*y*_/*d*_*x*_ = 2, which was estimated from the SEM image.

The optical transmittance was simulated by Rayleigh scattering^41^ and the Beer–Lambert law^42^ (Section 5 of Supplementary) with the nanopore volume fraction of 0.5% estimated above. When the diameter of the nanopores is larger than 40 nm, the transmission drops in the visible region (Fig. 4c). The transmission loss in the visible region significantly decreases with decreasing diameter from 40 to 10 nm. By comparing the measured (blue solid line in Fig. 1e) and simulated spectra, the diameters of nanopores for the low-loss modification could be estimated as between 20 and 30 nm. The average diameter of nanopores from the SEM image (Fig. 2a, *N*_p_ < 200) is ~30 nm, which shows a good agreement with the estimation.

Elucidation of the origin of the transmission loss and birefringence is crucial for the creation of practical birefringence in glass. Until now, the formation of periodic nanostructures or the alignment of lamellar structures has been believed to be necessary to generate birefringence by laser direct writing. However, the SEM observations and birefringence and transmittance simulations proved that spatially separated nanopores with anisotropic shape are sufficient to produce high enough birefringence with ultralow loss. Although both the birefringence and transmittance can be predicted by classical theories, this is the first demonstration of a new silica structure, which is very different from conventional nanoporous silica where pores aggregate^43^ or connect to each other^44^, and of space-selective control of the nanopore anisotropy with a focused polarized light beam.

### Mechanism of formation of anisotropic nanopores

The mechanism of polarization-controlled oblate nanopore formation should involve at least two processes: (1) formation of randomly distributed nanopores and (2) elongation of nanopores perpendicular to the polarization of the light beam. The formation of nanopores suggests the generation of oxygen molecules inside silica by laser irradiation, whose relation to defects formation has been investigated in a number of studies^35,45–48^. The fact that the low-loss birefringent modification (type X) cannot be observed with short pulse duration (*t*_p_ < 220 fs) (Fig. 3c) indicates that the formation of nanopores requires a time duration above a critical value. One possible reason is the interaction between the light pulse and transient defect pairs [E′ centre and NBOHC] or self-trapped triplet excitons (STE). Saeta et al. observed the generation of the transient defect pairs within ~250 fs after photoexcitation in silica glass^45^.

1 $$\equiv {\mathrm{Si}} - {\mathrm{O}} - {\mathrm{Si}} \equiv \mathop { \to}\limits^{250\;{\mathrm{fs}}} \equiv {\mathrm{Si}} \cdot + \cdot {\mathrm{O}} - {\mathrm{Si}} \equiv$$

where ≡ Si· is the E′ centre and ≡ Si–O· is the NBOHC. When the pulse duration is longer than 250 fs, the photoexcitation of the NBOHC could occur due to multiphoton absorption or impact ionization by hot electrons (~18 eV, double of the band gap of silica glass, 9 eV) produced by the avalanche mechanism, resulting in the dissociation of oxygen atom:

2 $$\equiv {\mathrm{Si}} \cdot + \cdot {\mathrm{O}} - {\mathrm{Si}} \equiv \mathop { \to }\limits^{{\mathrm{h}}\nu\;{\mathrm{or}}\;{\mathrm{e}}^- } \equiv {\mathrm{Si}}{ \cdot ^+ }{\mathrm{Si}} \equiv +\, {\mathrm{O}}^0 + {\mathrm{e}}^-$$

where ≡ Si·^+^Si ≡ is the E_δ_′ centre, O^0^ is an interstitial oxygen atom and e− is an electron. The dissociation of oxygen atoms from the silica structure is essential to generate oxygen molecules, which could facilitate nanopore formation in silica glass. For the laser pulse shorter than 250 fs, the probability of reaction (2) is reduced, resulting in the absence of nanopores for *t*_p_ < 220 fs. In contrast, at longer pulse durations, more oxygen atoms could be generated from reaction (2) by photoexcitation of the transient defect pairs. In addition, the contribution of avalanche ionization increases with increasing pulse duration, suggesting that the dissociation of the Si–O bond might be driven by high-energy electrons generated by avalanche ionization. The mechanism based on reaction (2) is supported by the absorption and photoluminescence spectra of modifications of different types (Section 6 of Supplementary), in which only the NBOHC is detected in the optically isotropic modification (type I), while both the oxygen-deficiency centers (ODCs) and NBOHC are detected in the birefringent modifications (type X and type II).

We measured the transmission of laser pulses during laser writing and found that 10–15% of the laser pulse energy was absorbed via photoexcitation. If all the absorbed light energy is used for heating the lattice of the glass, then the estimated temperature just after the photoexcitation is 1600–2100 K, which is high enough for cavitation in silica melt. On the other hand, the thermal quenching must be fast enough to avoid coalescence of nanopores. The viscosity of silica melt below the estimated temperature, 2100 K, is as high as 10^5^ Pa s, at which the diffusion coefficient of an oxygen molecule (diameter of 0.35 nm) is ~*D* = 9.2 × 10^−17^ m^2^/s according to the Stokes–Einstein equation^42^. At this diffusion coefficient, the expected diffusion length in *t* = 1 μs is (*D* × *t*)^1/2^ = 10 pm, which means no movement without any external force or no coalescence of nanopores by diffusion when the thermal quenching occurs within several microseconds.

For longer pulse durations, the transition from the low-loss to high-loss modification occurs with a smaller number of pulses (Fig. 3c and Section 1 of Supplementary). One of the effects of the longer pulse duration is an excess temperature increase due to the increased contribution of avalanche ionization^36,49^. Irradiation with a longer pulse could prevent the generation of spatially separated nanopores due to the higher temperature which facilitates the generation, growth and coalescence of nanopores.

Another essential process, elongation of nanopores in the direction perpendicular to the polarization, can be explained by the near-field enhancement around nanopores during laser irradiation^27,34^. The local electromagnetic field around a nanopore in a dielectric medium is enhanced perpendicular to the polarization. The enhanced field could induce more local ionization and generate an anisotropic stress distribution around the nanopore, which could cause its elongation in the direction perpendicular to the polarization (Section 7 of Supplementary, Fig. S6).

### Applications of low-loss birefringent modification

**Fig. 5:**
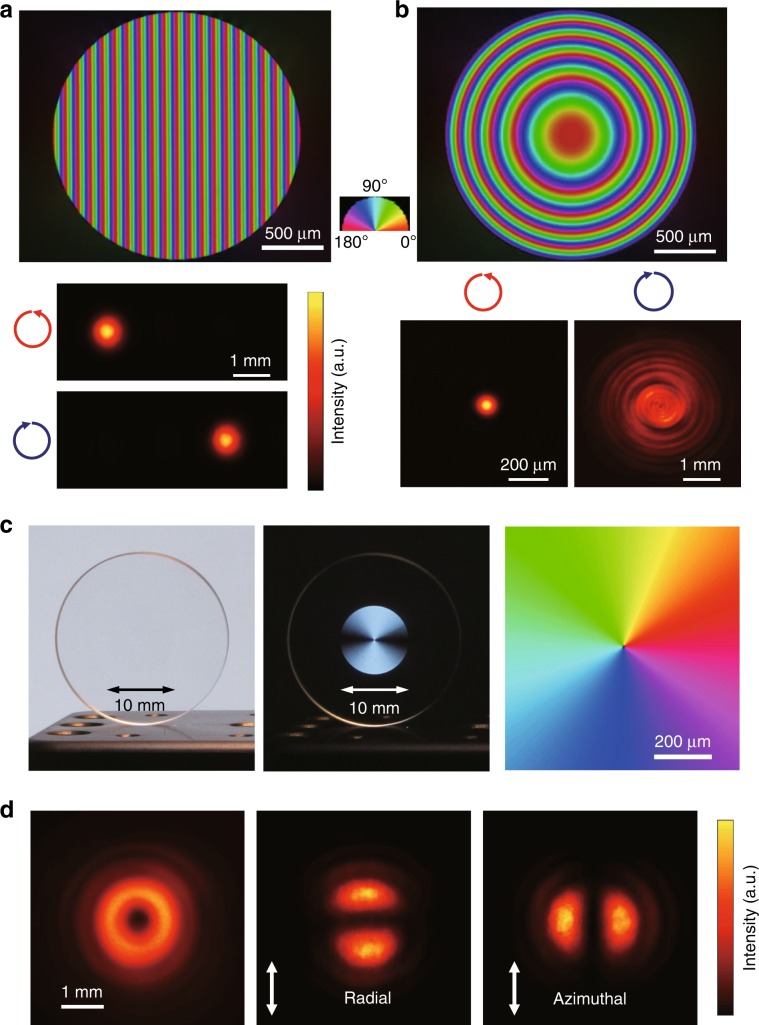
Geometric phase (GP) optical elements and vector beam converter imprinted with the low-loss birefringent modification. **a** Birefringence image of a GP prism with a slow axis gradient of 0.01 πrad μm^−1^ (upper), and light intensity patterns of 457 nm CW laser beams with different circular polarizations diffracted by the GP prism (lower). **b** Birefringence image of a GP lens, and intensity patterns of 488 nm CW laser beams with different circular polarizations focused and defocused by the GP lens. The focal lengths are ±208 mm for the wavelength of 488 nm. **c** Ten-millimetre vector beam converter without (left) and with (middle) a polarizer under linearly polarized white light illumination. The slow axis distribution in the central part of the converter is shown on the right. **d** Intensity pattern of a 343 nm laser beam after the converter without a polarizer (left). Intensity patterns of the radial (middle) and azimuthal (right) vector beams produced by the converter after a polarizer

The low-loss birefringent modification provides a variety of birefringent optical elements, such as GPOEs, vector beam convertors, and true zero-order waveplates. The fabricated GP prism or polarization grating has a birefringence distribution with a constant slow axis gradient along the horizontal direction (Fig. 5a). This allows continuous phase shifts without phase resets, in contrast to conventional optical elements, such as blazed gratings and Fresnel lenses, wherein the phase profiles are recorded as optical path variation in the refractive index and thickness. Moreover, the direction of light propagation can be switched by changing the handedness of the circular polarization of incident light (Fig. 5a). The demonstrated diffraction efficiency was higher than 99% at 457 nm, which agrees with that calculated for the measured retardance of 220 nm. A GP lens with a parabolic shape of the slow axis distribution was also fabricated (Fig. 5b). The focusing and defocusing of the GP lens can be switched by changing the handedness of the circular polarization. A 488 nm laser beam was focused to the diffraction-limited spot size of 112 μm, close to the theoretical value of 114 μm. An F-number as small as 50 was demonstrated, which is limited by the phase gradient of ~0.1 π rad/μm. Interestingly, the same highly transparent GP lens in glass could act as an all-in-one concave–convex lens for the correction of short- and long-sightedness.

Another important application is a polarization vector beam converter (Fig. 5c). In the converter, the retardance was chosen to as half of the target wavelength (343 nm) and the slow axis was linearly varied from 0° to 180° with respect to the azimuth (Fig. 5c right). The imprinted beam converter was highly transparent (Fig. 5c left), while it could be clearly observed under cross-polarizers (Fig. 5c middle). A high-quality 343 nm dounut-shaped beam with radial and azimuthal polarization was generated (Fig. 5d). Transmittance through the beam converter as high as 91% was measured without any evidence of damage for the 343 nm laser beam with an average power of 1.2 W, beam width of 4 mm, pulse duration of 190 fs and repetition rate of 1 MHz.

In summary, we observed ultrafast laser-induced modification in silica glass with the evidence of anisotropic nanopore formation representing a new type of nanoporous material. The modification enabled fabrication of ultralow-loss birefringent optical elements including geometrical phase elements, vector beam converters and zero-order retarders, which can be used for high power lasers and UV light sources. The high transmittance from the UV to near-infrared, high damage threshold and thermal resistance of the fabricated optical elements in silica glass overcome the limitations of GP and polarization shaping using other materials including liquid crystals and meta-surfaces. The space-selective birefringent modification with high transparency also enables high density multiplex data storage in silica glass^50^.

## Materials and methods

### Laser writing of birefringence

The writing of birefringent modifications in silica glass was carried out with a PHAROS Yb-doped potassium gadolinium tungstate (Yb:KGW)-based mode-locked regenerative amplified femtosecond laser system (Light Conversion Ltd.) operating at 1030 nm with a variable repetition rate from 1 kHz to 1 MHz and a pulse duration from 190 fs to 10 ps. The laser beam was focused via a 0.16 NA aspheric lens into silica glass plates (Viosil, ShinEtsu, OH content 1200 ppm) mounted on a computer-controlled three-axial air-bearing translation stage (Aerotech ABL1000) (Fig. 1a). The diameter of the laser beam at the focus was estimated to be ~5 μm. For writing of birefringent patterns, parallel birefringent lines were written by raster scanning with a line separation of 1 μm by translating a silica glass plate perpendicular to the incident laser during laser irradiation at a repetition rate (*υ*_p_) of 200 kHz. The slow axis distribution of the birefringence was controlled by changing the polarization azimuth of the writing beam using a combination of a polarizer, an elecro-optic modulator (EOM) and a quarter waveplate (QWP). The polarization azimuth of the writing beam was controlled by the voltage applied to the EOM (Section 8 of Supplementary). The pulse densities (*N*_d_) during raster scanning were controlled by changing the scanning speed (*v*_s_). The pulse density was defined by *N*_d_ = *υ*_p_/*v*_s_. To obtain the required retardance value for GPOEs and vector beam converters, multiple birefringent layers were written with a layer separation of ~40 μm.

### Characterization of laser written structures

Birefringence measurement of imprinted structures was carried out with BX51 (Olympus) optical microscope equipped with a quantitative birefringence measurement system (Abrio; CRi. Inc.) operating at 546 nm. Optical transmission images were obtained by removing the colour filter and polarization optics from the microscope. Phase imaging for refractive index measurement was carried out with a wavefront sensor camera (SID4; PHASICS) equipped on the same microscope. Transmission spectra were measured with Cary 500 UV–VIS–NIR spectrometer.

For observation of the nanostructures, the laser-processed glass was lapped and polished in the modified region and the exposed surface was etched with a 1 mol/L KOH solution in 24 h. Imaging of the etched surfaces was performed with a scanning electron microscope (SEM, Zeiss EVO 50).

## Supplementary information

Supplemental material
