# Supplementary material for: Ultralow-loss geometric phase and polarization shaping by ultrafast laser writing in silica glass
Source: Light Sci Appl. 2020 Feb 4;9:15. doi: 10.1038/s41377-020-0250-y (PMC7000703; doi:10.1038/s41377-020-0250-y)
Supplement: Supplementary file 1 — Supplemental material [file 41377_2020_250_MOESM1_ESM.docx]

**Supplementary information for**

**Ultralow-loss geometric phase and polarization shaping by ultrafast laser writing in silica glass**

Masaaki Sakakura1,*, Yuhao Lei1, Lei Wang1,**, Yan-Hao Yu1, and Peter G Kazansky1

*1Optoelectronics Research Centre, University of Southampton, Southampton, SO17 1BJ, United Kingdom*

Correspondence: [M.Sakakura@soton.ac.uk](mailto:M.Sakakura@soton.ac.uk), [L.Wang@soton.ac.uk](mailto:L.Wang@soton.ac.uk)

1. **Modification types with different writing parameters**

The clarification of the relation between laser writing parameters and modification types is a key to understand the mechanism. We characterized the modification types using the birefringence and transmission of laser written areas on pulse duration (*tp*), pulse energy (*Ep*) and pulse density (*Nd*). The areas of 20 µm × 10 µm were written by raster scanning with a 1 µm pitch between lines by focusing laser beam inside silica glass with a 0.16 NA aspherical lens. The lines were written with different scanning speed (*v*s) for each area. The pulse density was calculated by *v*/*v*p, where *v* is the translation speed and *v*p is the repetition rate of 200 kHz.

**
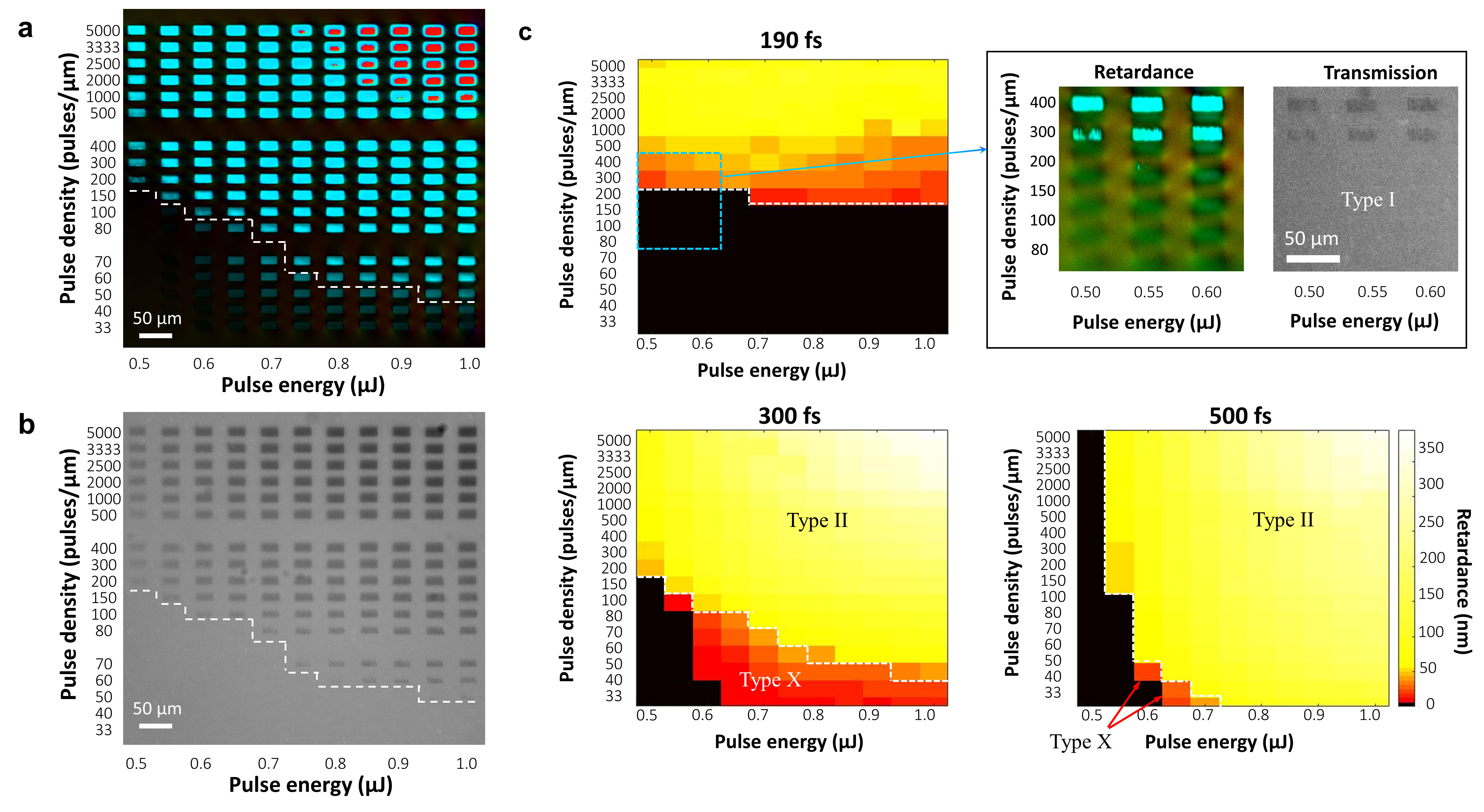
**

**Fig. S1 a, b** Birefringence and transmission images of laser writing areas with tp=300 fs, different pulse energy and different pulse densities, respectively. **c** Retardance maps as functions of pulse energy and pulse density at different pulse durations. The inset beside the retardance map of tp=190 fs shows retardance and transmission images of modification areas written by 190 fs laser pulses. The white dashed lines indicate the boundary between "invisible" and "visible" modification.

The modification types were distinguished from the birefringence and transmission images [Fig. S1**a** and S1**b**, respectively]. By comparing the birefringence and transmission images, several birefringent structures are visible (type II) while others cannot be seen (type X) in the transmitted light. For tp=190 fs, the appearance of retardance is accompanied by the transition from invisible to visible modifications [Fig. S1**c**]. Although the invisible areas had no clear birefringence (retardance of <2 nm), they can be observed by the stress-induced birefringence [the inset of Fig. S1**c**], which is due to isotropic density change in silica structure (type I). For tp=300 fs, invisible birefringent modification was generated with moderate pulse energy and smaller pulse density. For tp=500 fs, the writing parameter range of invisible birefringent modification becomes smaller compared with that at tp=300 fs, and no invisible birefringent modification was observed with tp=700 fs.

1. **Birefringence of laser induced modification**

The difference between refractive indices for ordinary and extraordinary waves (Δnb=|ne-no|) was estimated by the measured retardance (Δb) and the length of the birefringent structure (*l*) along light propagation axis [Fig. S2a] by the relation:

Δnb =|ne-no|= Δb /*l* (S1).

The birefringent structures were written by raster scanning with 300 fs, 0.7 µJ, 1030 nm laser pulses at 200 kHz at different pulse densities. Based on the measured transmittance (T550 nm), the birefringence of low-loss birefringent modification (T550 nm>99 %) is smaller than about 6×10-3 and that of high-loss one (T550 nm < 90 %) is 2-3×10-3.


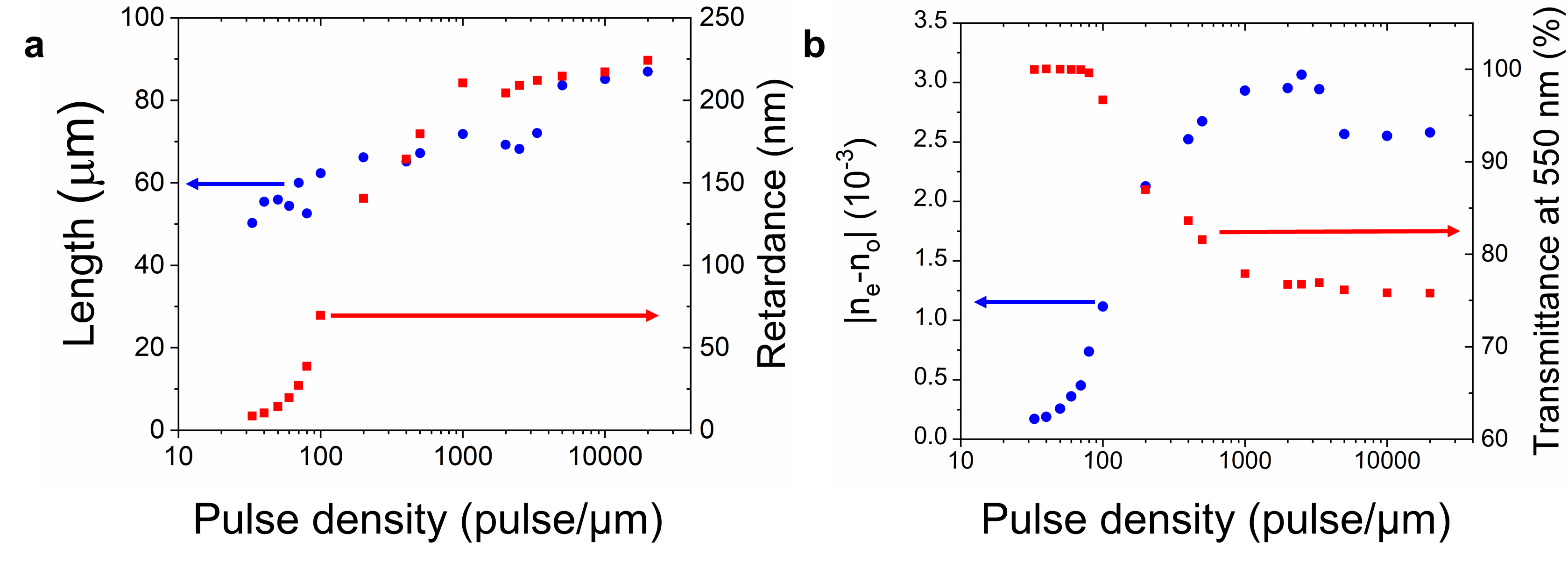


**Fig. S2** **a** Measured length and retardance of the birefringent structures, **b** Birefringence calculated by the retardance and length and transmittance at different pulse densities.

1. **Refractive index measurement of laser written region**

The refractive index change of laser written regions was measured by the phase imaging with transmission light of different polarization states (Fig. S3**a**). The regions of 100 μm × 20μmm were written by vertically (90º) polarized laser pulses of different pulse energies, which generated birefringent modifications of slow axis of 0º. The refractive index change was calculated by the phase image and the axial length of the laser written region (50 μm, Fig. S2**a**). The refractive index profile along the dashed line in Fig. S3**a** shows that the positive refractive index change at shorter pulse duration (Δnunpol~5×10-4) becomes smaller with increasing pulse duration (Fig. S3**b**). The refractive index change became from positive to negative at the pulse duration of 350 fs (Fig. S3**c**), at which the stress in the vicinity of the modified region was the smallest (Fig. 3**e** and 3**f**). The decrease of the refractive index indicates that the number of nanopores increased with increasing pulse duration. In addition, the difference between Δn0° and Δn90° increases with increasing pulse duration, indicating the increase of birefringence (Fig. S3**d**). The small birefringence (2×10-4) at shorter pulse duration is stress-induced birefringence, which does not depend on the polarization of the writing beam. The birefringence measured by the phase imaging (Δn=3×10-4 at 300 fs) is almost consistent with that by the birefringence imaging (Δn=5×10-4, Fig. S2**b**).

**
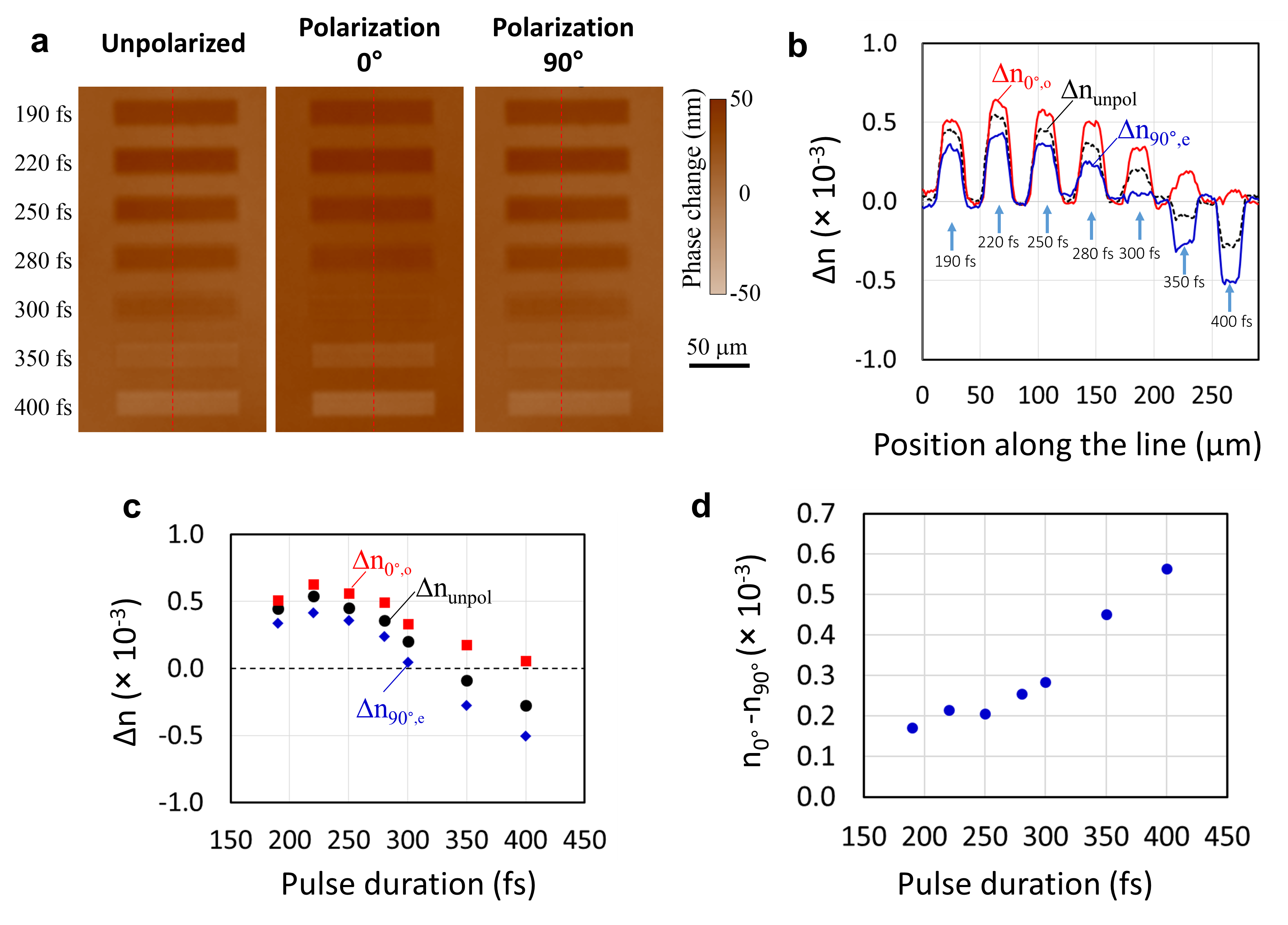
**

**Fig. S3.** Measurement of the refractive index change in the laser written regions. **a** Phase images of laser written regions at different pulse durations. Three phase images were obtained with unpolarized light, horizontally (0º) and vertically (90º) polarized light. **b** The refractive index changes for different polarization states (Δnunpol, Δn0° and Δn90°) along the dashed lines in **a**. **c** The refractive index changes for different polarization states at different pulse durations. **d** Δn0°-Δn90° (birefringence) at different pulse durations.

1. Low-loss birefringent modification with different numerical aperture

We found that the formation of low-loss birefringent modification becomes more difficult with higher numerical aperture (NA). For the pulse duration at 300 fs, the transition from the low- (Type X) to high- (Type II) loss modifications occurs at smaller number of pulses (Np) with higher NA (Np=200 with NA=0.16, Fig. S4a; Np=120 with NA=0.25, Fig. S4b). In addition, the highest retardance for the Type X is larger with smaller NA (25 nm with NA=0.16, 15 nm with 0.25). With the numerical aperture higher than 0.30, no Type X modification has been observed, indicating that the difference cannot be explained only by different photoexcited volume with different NA.


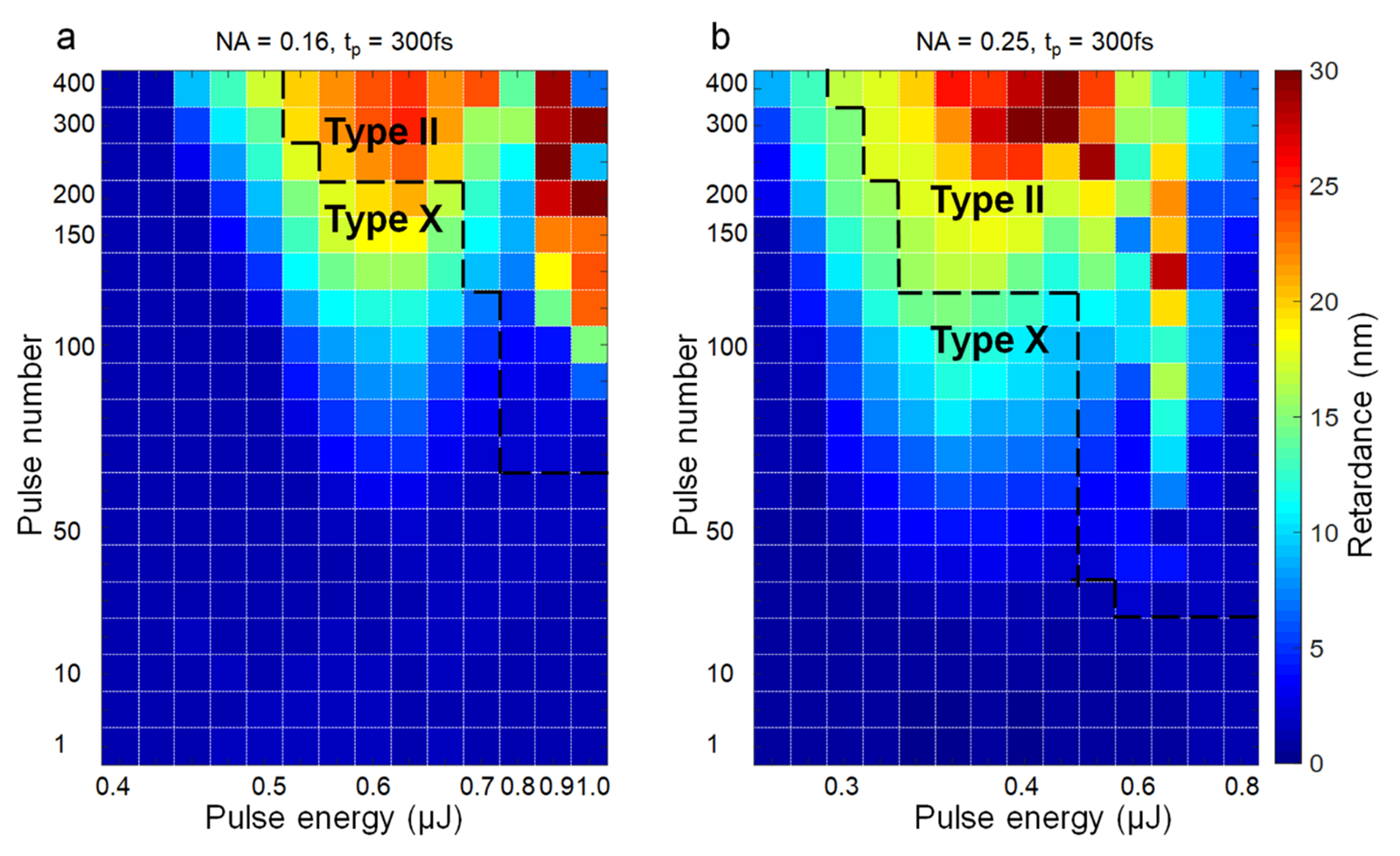


Fig. S4. a, b Retardance maps as functions of pulse energy and pulse number with numerical apertures of 0.16 and 0.25 for the focusing lens, respectively. The dashed lines are the boundary between laser writing conditions for low- (Type X) and high- (Type II) loss modifications.

1. Calculation of birefringence and transmission

The birefringence originated from elongated nanopores was simulated by the equation based on Maxell-Garnett model with anisotropic scattering particles. For simplicity, it is assumed that the oblate nanopores of the identical shape are distributed in silica glass [Fig. 3a]. Based on the formula derived by Sihvola and Kong[[1]](#endnote-1), the effective permittivity of silica containing oblate nanopores inside can be expressed by

(S2)

where ε and  are the dielectric constants of silica glass and vacuum (=*n*2=2.1), respectively, *n* is the refractive index of silica glass, *f* is the volume fraction of spheroid nanopores inside silica, and *N*i is the depolarization factor of the oblate nanopore. The depolarization factor can be written as

(S3)

and *N*y=1-2*N*x, where *d*x and *d*y are the lengths of the nanopore along *x* and *y* axes, respectively. After getting the depolarization factors, the effective permittivities, and were calculated using eq. (S2) and the birefringence (nyy-nxx) was calculated by

(S4).

The transmission loss by nanostructures is produced by Rayleigh scattering. The scattering cross section can be written by[[2]](#endnote-2)

(S5)

where *d* is the diameter of nanopore, *nm*  is the refractive index of the material and  is the wavelength of the scattered light. Once the cross section was calculated, the light intensity after passing through the material was calculated by Beer-Lambert law. When the number of nanopores per unit volume is , the light intensity is:

(S6)

where *L* is the thickness of the medium with nanopores, and *I*0 and *I*(*L*) is the light intensity before and after the material respectively.

1. UV-VIS absorption and photoluminescence spectra of laser induced modification in silica glass

The formation of defects in laser induced modification inside silica glass was investigated by measuring the UV-VIS absorption and photoluminescence spectra. To measure the spectra, structures with a diameter of 5 mm were written at the depth of 0.2 mm inside a silica glass plate by raster scanning with laser pulses of 0.7 μJ at 1030 nm focused with a 0.16 NA aspheric lens. Other writing parameters for different modifications are listed in Table S1. For comparison, we fabricated both type X and type II with same retardance level (~150 nm), which means seven layers type X and a single layer type II. In addition, seven layers type I modification has been constructed for further measurements.

|  | **Pulse duration** | **Scanning speed** | **Retardance (Single layer)** | **Transmittance at 550 nm** |
| --- | --- | --- | --- | --- |
| **Isotropic modification (Type I)** | 190 fs | 6 mm/s |  | 99 % |
| **Low-loss birefringent modification (Type X)** | 300 fs | 6 mm/s | 20 nm | 99 % |
|  | 500 fs | 8 mm/s | 46 nm | 97 % Ref.3 |
| **High-loss birefringent modification (Type II)** | 500 fs | 1 mm/s | 186 nm | 67 % |

**Table S1** Different laser writing parameters for three modification types (Type 1: low-loss isotropic modification; Type 2: high-loss birefringent modification; Type X: low-loss birefringent modification).

The UV-VIS absorption spectra were measured with a Cary 500 UV-VIS-NIR spectrometer. The absorption spectra reveal several absorption peaks [Fig. S5**a**], which show the generation of different defects in silica glass after laser writing, in contrast to no obvious absorption peaks from 200 nm to 800 nm in the pristine silica glass. The absorption bands assigned to E' centers at 210 nm (≡Si, an unpaired electron in a silicon atom bound to three oxygen atoms) were observed in all the modifications, while those assigned to ODC(II) centers (-O-Si-O-, a divalent silicon atom) or Eδ' center (≡Si·+Si≡) around 245 nm were observed only in birefringent modifications. The absorption bands of non-bridging oxygen center (~620 nm) were observed clearly in low-loss modifications, but it is not clear in high-loss modification due to the high transmission loss. The photoluminescence spectra excited by 343 nm light [Fig. S5**b**] measured with a ANDOR SR-3031-A spectrometer reveal the luminescence bands of NBOHC (~650 nm) in all the modifications as well as 540 nm emission observed only in birefringent modifications (Type II and Type X) by ODC defect centers, possibly Eδ' center4,5. The small absorption band around 620 nm caused by NBOHC could be quenched by annealing at 400 ºC for two hours (Fig. S5**c**).


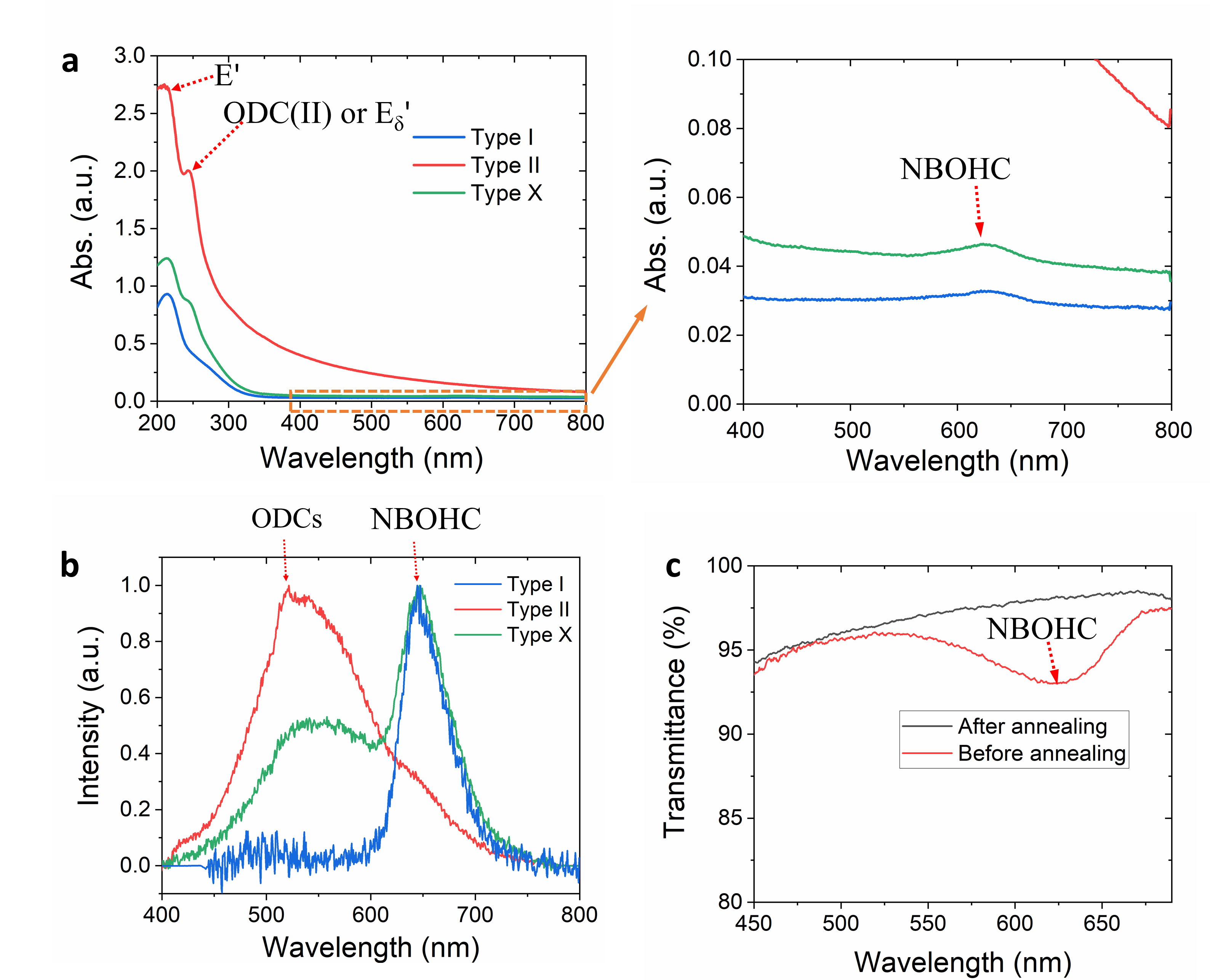


**Fig. S5** **a** UV-VIS absorption spectra of different modifications in silica glass; **b** Photoluminescence spectra of different modifications with 343 nm laser excitation. **c** The absorption of the NBOHC before after the annealing at 400 ºC for two hours.

1. Simulation of local enhanced field around a nanopore

The electric field around a nanopore in silica glass during laser irradiation was simulated by a Finite Element Method (FEM) using COMSOL Multiphysics (COMSOL Inc.). In the simulation, and a linearly polarized plane electromagnetic wave at 1030 nm in vacuum was incident on a nanopore with a diameter of 20 nm inside silica glass (black circle at the center in Fig. S6). For simplicity, the stationary solution of the continuous electromagnetic wave was calculated. The calculated intensity of the electromagnetic wave shows a light field enhancement along the horizontal direction (perpendicular to the polarization direction) with a factor of more than 1.8. As discussed in the paper, the enhanced field could induce the directional deformation of the nanopore perpendicular to the polarization.


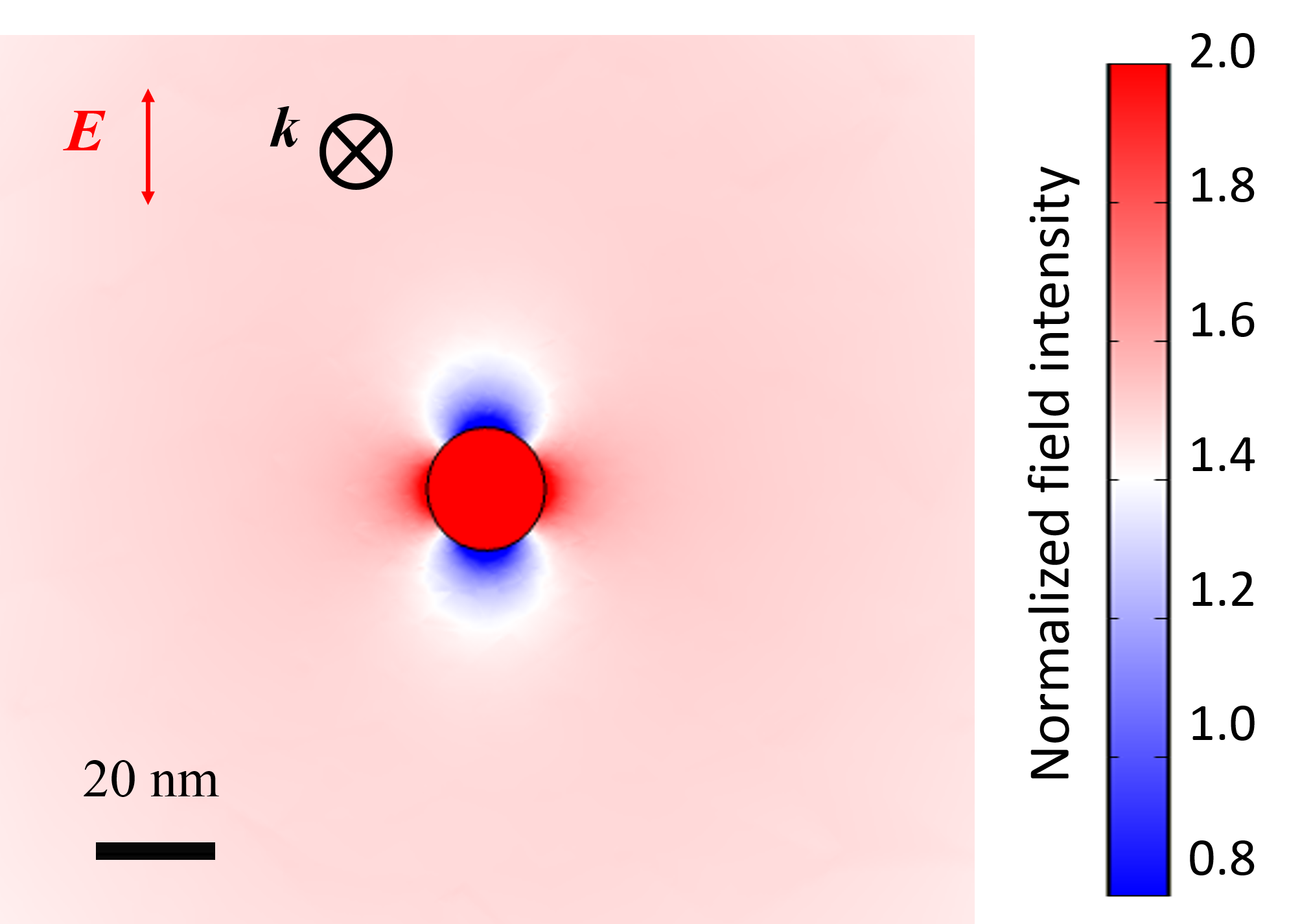


**Fig. S6.** The light intensity distribution around a nanopore inside silica glass by the simulation. The red arrow represents the polarization direction of the incident light. The light propagates perpendicular to the plane of the picture. The scale bar denotes the normalized light field enhancement factor.

1. **Polarization control with an EOM**

In the laser writing setup (Fig. 1**a**), the orientation of the linear polarization of a writing beam is controlled with a linear polarizer, an electro-optic modulator (EOM) and a quarter waveplate (QWP). The slow axis of the QWP is parallel to the orientation of the polarizer. The optical axis of the EOM is oriented at 45º to the polarization. Here, the Stokes vector of the writing beam after the polarizer is defined as

(S.7)

The Mueller matrices of the QWP and EOM are given respectively by

(S.8)

(S.9)

, where  is the retardance given by the EOM, which can be controlled by the applied voltage to the EOM. Therefore, the polarization after the EOM and QWP is calculated by

(S.10)

,which indicates that the orientation of the linear polarization can be controlled by the applied voltage to the EOM.

**References**

1. . Sihvola, A. H. & Kong, J. A. Effective Permittivity of Dielectric Mixtures. *IEEE Trans. Geosci. Remote Sens.* **26**, 420-429 (1988). [↑](#endnote-ref-1)
2. . Cox, A. J., DeWeerd, A. J. & Linden, J. An experiment to measure Mie and Rayleigh total scattering cross sections. *Am. J. Phys.* **70**, 620-625, (2002).

   3. Drevinskas, R., Kazansky, P., Cerkauskaite, A. Nanostructured optical element, method for fabrication

   and uses thereof. (2019).

   4. Mishchik, K., d'Amico, C., Velpula, P. K., Mauclair, C., Boukenter, A., Ouerdane, Y., & Stoian, R. Ultrafast laser induced electronic and structural modifications in bulk fused silica. *J. Appl. Phys.* **114**, 133502 (2013).

   5. Nishikawa, H., Watanabe, E., Ito, D., Sakurai, Y., Nagasawa, K., & Ohki, Y. Visible photoluminescence from Si clusters in γ‐irradiated amorphous SiO2. *J. Appl. Phys.* **80**, 3513-3517 (1996). [↑](#endnote-ref-2)
